# Medical Ethics Awareness Among Doctors at the Pakistan Institute of Medical Sciences

**DOI:** 10.7759/cureus.112991

**Published:** 2026-07-20

**Authors:** Muhammad Anas Waheed Jami, Nida Khan, Saniya Siddique, Fibhaa Syed, Mohammad Ali Arif, Shafat Khatoon

**Affiliations:** 1 Internal Medicine, Pakistan Institute of Medical Sciences, Islamabad, PAK; 2 Internal Medicine, Shaheed Zulfiqar Ali Bhutto Medical University, Islamabad, Islamabad, PAK

**Keywords:** bioethics, doctor – patient, ethical awareness, ethical dilemmas, ethics education, medical ethics, pakistan, pims (pakistan institute of medical sciences

## Abstract

Objectives

This study aims to determine the level of medical ethics awareness among doctors working at a tertiary care hospital and the factors related to adequate awareness.

Methods

A cross-sectional descriptive study was conducted over a period of three months at the Pakistan Institute of Medical Sciences (PIMS), Islamabad. The study used a complete enumeration approach to include all eligible doctors who met the inclusion criteria, i.e., house officers, medical officers, postgraduate trainees, and consultants. A structured questionnaire, originally developed as the Omani Physicians Bioethics and Medical Law Awareness Tool and subsequently adapted and used in a Pakistani healthcare setting, was used for data collection. The questionnaire assessed awareness of ethical dilemmas and specific issues in medical ethics. IBM SPSS Statistics for Windows, Version 25 (Released 2017; IBM Corp., Armonk, New York) was used to analyze the data. Descriptive statistics were calculated, and the associations between ethical awareness and the study variables were assessed using appropriate statistical tests. A p-value of less than 0.05 was taken as statistically significant.

Results

A total of 265 doctors participated in our study. Overall, 64.5% of the participants showed adequate ethical awareness. The mean score for awareness of ethical dilemmas was 9.6 ± 2.8 out of a maximum of 14, while the mean score for awareness of specific issues in medical ethics was 27.4 ± 5.6 out of 40. Significantly higher ethical awareness was observed among senior doctors and participants with prior ethics training, whereas no significant association was found between ethical awareness and gender.

Conclusion

Most doctors exhibited a satisfactory baseline of ethical awareness, yet critical vulnerabilities remain among junior cohorts and those without prior training. To bridge these gaps, medical institutions must implement continuous, structured, and case-based ethics training within undergraduate curricula and mandatory clinical rotations. Standardizing mandatory ethics modules prior to clinical residency is essential to build foundational moral resilience early in medical careers.

## Introduction

Biomedical ethics is a key component of healthcare, encompassing ethical, legal, and social dimensions of medical practice [1]. Biomedical ethics provides a schema for resolving challenging moral dilemmas that occur in clinical service, research, and health policy. The discipline of biomedical ethics is guided by four principles: autonomy, beneficence, non-maleficence, and justice [2]. These principles assist caregivers in making sure their care is rooted in integrity by respecting the rights and dignity of patients.

Ethical behavior is fundamental to all aspects of professional medical practice [3]. Expectations of healthcare professionals are to maintain their professionalism, which encompasses providing patient-centered care that promotes both physical and mental well-being [4]. In their everyday practice, healthcare workers (HCWs) are facing ethical dilemmas on a daily basis that may include not obtaining informed consent from patients, breach of confidentiality, withholding information from patients, conflicts of interest (e.g., receiving gifts from pharmaceutical companies), or boundary violations with patients [5].

Although global attention is being paid to the ethics of medical practice, there is a worrying trend of unethical behavior among healthcare workers, including in Pakistan. Legal actions are being taken against healthcare providers and professionals in response to unethical practices, indicating public awareness and concern regarding ethical misconduct [6]. The Pakistan Medical and Dental Council (PMDC) has developed a code of medical ethics specific to the local culture and profession; however, the lack of formal teaching and practice of ethics in medical education is still a major issue.

The lack of formal teaching is evident at both general medical education and postgraduate training levels, with ethics education often minimal [7]. The health care system is currently under immense strain due to high patient volumes and increasing procedural complexities, making ethical dilemmas more frequent and increasingly difficult to deal with. Ethical issues not only harm the doctor-patient relationship when left unaddressed, but they also jeopardize patient safety and increase the risk of aggression and/or litigation [8].

Medical ethics is an essential component of medical practice, ensuring professionalism and patient-centered care. A cross-sectional study conducted among dentists in Nineveh Governorate demonstrated that medical ethics awareness varied significantly according to professional designation, with specialists exhibiting substantially higher knowledge levels (98.7%) than general practitioners (80%) and interns (55%), whereas no significant association was observed between ethics knowledge and gender. These findings suggest that professional experience and clinical seniority may contribute to greater ethical awareness [9].

A study conducted in Khyber Pakhtunkhwa among HCWs reported that doctors were more likely than other participants to encounter ethical situations and commonly relied on personal experience or senior guidance when addressing them. Consultants also reported observing unethical decisions more frequently (51.1%) than other professional groups. Significant associations with professional designation were observed for several ethically sensitive practices, such as accepting gifts from patients (p=0.002), receiving remuneration for patient referrals (p<0.001), incomplete disclosure of medical errors (p=0.008), and advising patients to use company-specific products (p<0.001) [10].

Although the study conducted in Khyber Pakhtunkhwa provided useful insight into medical ethics awareness among healthcare professionals in Pakistan, it was conducted across private hospitals, basic health units, rural health centers, and district-level hospitals, which differ substantially from tertiary care teaching hospitals in terms of clinical complexity, patient load, and exposure to ethical dilemmas. Furthermore, its findings may not fully reflect the demographic and institutional diversity seen in Islamabad, where patients are referred from across the country and physicians represent a more heterogeneous professional and cultural background. The Pakistan Institute of Medical Sciences (PIMS) is a major tertiary care teaching hospital serving as a national referral center. Despite its importance, evidence regarding physicians' awareness of medical ethics in large public-sector tertiary care hospitals in the federal capital remains scarce.

Therefore, this study was undertaken to evaluate the level of medical ethics awareness among doctors working at PIMS, Islamabad, and to explore factors that may be associated with ethical awareness, including professional designation, gender, and prior exposure to ethics training.

## Materials and methods

Study design

A cross-sectional descriptive study was conducted at the Pakistan Institute of Medical Sciences (PIMS), Islamabad.

Target population

The study was conducted among doctors currently working at PIMS, such as house officers, medical officers, postgraduate trainees, and consultants.

Inclusion criteria

The study included all actively working doctors with a minimum of six months of clinical experience in the current setting who provided informed consent to participate.

Exclusion criteria

Laboratory technicians, radiologists, medical or nursing students, administrative people, or researchers were excluded from the study. Doctors who do not regularly interact with the patients or were on long leave or absent during the data collection period were also excluded.

Sampling technique and sample size

The study used a complete enumeration approach to include all eligible doctors working at PIMS who met the inclusion criteria, ensuring comprehensive coverage of the target population. A total of 265 participants were identified as eligible and invited to participate in the study. The study achieved a response rate of 100%, and all eligible participants were included in the final analysis.

Study duration

The study was conducted over a total duration of three months, starting from the date of approval by the Ethical Review Research Committee (ERRC) of PIMS.

Data collection

The data collection tool was a structured questionnaire originally developed as the Omani Physicians’ Bioethics and Medical Law Awareness Tool and subsequently adapted and used in a Pakistani healthcare setting [10,11]. In that study, the instrument demonstrated acceptable internal consistency (Cronbach’s alpha = 0.659). The present study adopted this previously used version without modification to the validated core items assessing ethical knowledge and related domains. Only minor contextual adjustments were made to the demographic section to align with the study population and setting (Appendix 1). The questionnaire comprised 34 closed-ended items covering general information, legal knowledge, ethical practice, responses to ethical dilemmas, and selected issues related to medical ethics. For ease of administration and wider reach, the questionnaire was converted into a self-administered Google Form. It was distributed through official institutional communication channels, and settings were applied to limit responses to one per email address to minimize the risk of duplicate submissions. Reminder messages were sent to non-responding participants during the data collection period to enhance participation.

Data analysis

Data were exported from Google Forms into IBM SPSS Statistics for Windows, Version 25 (Released 2017; IBM Corp., Armonk, New York) for analysis. For awareness regarding ethical dilemmas, every correct answer was given a score of two points, and every incorrect answer was given zero points, for a total of seven questions. Total awareness scores out of 14 were calculated. Similarly, for 10 questions assessing awareness regarding specific issues in medical ethics, each response was scored on a scale of 1-4, with the highest score awarded to the most appropriate answer. Participants’ awareness was evaluated, yielding a total score out of 40. Participants’ awareness was categorized as adequate if they correctly answered ≥70% of the related questions, while scores <70% were classified as inadequate. This cutoff was used to ensure consistency in scoring and interpretation and is in line with commonly applied thresholds in published KAP studies in healthcare research, where a 70% cutoff is frequently used for knowledge categorization [12,13].

Descriptive statistics were used to summarize the data. Categorical variables such as gender, designation, years of clinical experience, prior ethics training, and awareness categories were presented as frequencies and percentages. Awareness scores were reported as mean ± standard deviation (SD).

Associations between awareness levels (adequate versus inadequate) and participant characteristics were initially assessed using the Pearson chi-square test. Binary logistic regression was performed to estimate the association between participant characteristics and adequate ethical awareness. The results were reported as odds ratios (ORs) with their corresponding 95% confidence intervals (95% CIs). For comparisons of responses to individual ethical issues across professional designations, Pearson chi-square tests were used, and Cramer's V was calculated to assess the strength of statistically significant associations. A p-value <0.05 was considered statistically significant.

Ethical considerations

Data collection commenced after acquiring ethical approval from the Ethical Review Research Committee (ERRC) of PIMS (ERRC# F-5-2/2024-ERRC/PIMS). Informed consent was obtained digitally from all the participants before accessing the questionnaire. Confidentiality and anonymity were ensured throughout the research process.

## Results

The study involved a total of 265 doctors, including house officers, medical officers, postgraduate trainees, and consultants from different clinical departments of PIMS. More than half of the participants were male, and postgraduate trainees constituted the largest proportion of the professional groups. Almost two-thirds of the respondents reported having attended some form of ethics training previously (Table 1).

**Table 1:** Demographic characteristics (n=265)

Variable	Frequency (N)	Percentage (%)
Overall	265	100
Gender		
Male	154	58.1
Female	111	41.9
Designation		
House Officers	72	27.2
Medical Officers	64	24.2
Postgraduate Trainees	89	33.6
Consultants	40	15.1
Prior Ethics Training		
Yes	168	63.4
No	97	36.6

Overall, 171 (64.5%) doctors had adequate ethical awareness, and 94 (35.5%) had inadequate awareness. A statistically significant association was observed between overall awareness levels and professional designation as well as prior ethics training. A greater proportion of consultants and postgraduate trainees demonstrated adequate awareness compared to house officers and medical officers. Participants who had received prior ethics training demonstrated significantly better awareness than those who had not. No statistically significant association was found between gender and awareness level (Table 2).

**Table 2:** Association between ethics awareness levels and selected variables Data are presented as n (%). Associations between categorical variables and ethics awareness were assessed using Pearson's chi-square test for gender and prior ethics training. For designation, univariate logistic regression was performed using House Officers as the reference category; the reported test statistic represents the likelihood-ratio chi-square statistic for the overall model. Odds ratios (OR) with 95% confidence intervals (CI) are presented for logistic regression analyses. *Indicates a statistically significant p-value (<0.05) for the overall model or a significant difference compared to the reference category.

Variable	Adequate Awareness n (%)	Inadequate Awareness n (%)	Test Statistic	p-value	OR (95% CI)
Overall (N=265)	171 (64.5)	94 (35.5)	-	-	-
Gender			1.5	0.229	-
Male (N=154)	104 (67.5)	50 (32.5)	-	-	1.37 (0.82–2.27)
Female (N=111)	67 (60.4)	44 (39.6)	-	-	Reference
Designation			8.9	0.031*	-
House Officers (N=72)	39 (54.2)	33 (45.8)	-	-	Reference
Medical Officers (N=64)	39 (60.9)	25 (39.1)	-	-	1.32 (0.67–2.62)
Postgraduate Trainees (N=89)	63 (70.8)	26 (29.2)	-	-	2.05 (1.07–3.93)*
Consultants (N=40)	30 (75.0)	10 (25.0)	-	-	2.54 (1.08–5.96)*
Prior Ethics Training			19.6	<0.001*	-
Yes (N=168)	125 (74.4)	43 (25.6)	-	-	3.22 (1.90–5.47)*
No (N=97)	46 (47.4)	51 (52.6)	-	-	Reference

The mean awareness score for ethical dilemmas was 9.6 ± 2.8 out of a maximum possible score of 14. No significant difference in mean scores was observed between male and female participants, with male participants having a mean score of 9.43 ± 2.9 and female participants 9.84 ± 2.6. In contrast, participants who had received prior ethics training demonstrated significantly higher mean awareness scores than those without prior training, with mean scores of 10.15 ± 2.6 and 8.65 ± 2.9, respectively. Responses to individual ethical dilemma statements did not differ significantly across professional designations.

Considerable consensus was observed around some of the basic principles of ethics. These issues included the need to obtain the consent of the parents before conducting any treatment on the child, where 210 (79.2%) participants agreed, as well as the duty to serve inaccessible and disadvantaged communities, where 231 (87.3%) participants agreed. Likewise, 195 (73.6%) respondents agreed that close relatives should be informed of the status of their patients' health conditions. Some disagreement was observed for issues that are more complicated ethically, such as the doctor's authority during disagreements between the patient and his family over treatment plans, where 160 (60.4%) participants agreed, and refusal to conduct an abortion despite its legal permissibility, where only 120 (45.3%) participants agreed with the statement. However, 225 (84.9%) participants were opposed to assisting a patient in ending their life, regardless of their illness (Table 3).

**Table 3:** Responses to ethical dilemma statements according to professional designation N = total number of participants in each professional group; values in cells are presented as n (%). Pearson's chi-square test was used to compare responses across professional designations. p<0.05 was considered statistically significant.

Statement	House Officers (N=72)	Medical Officers (N=64)	PG Trainees (N=89)	Consultants (N=40)	Overall (N=265)	χ²	p-value
Children should not be treated without the consent of their parents						0.027	0.999
Agree	57 (79.2%)	51 (79.7%)	70 (78.7%)	32 (80.0%)	210 (79.2%)	-	-
Disagree	15 (20.8%)	13 (20.3%)	19 (21.3%)	8 (20.0%)	55 (20.8%)	-	-
Close relatives should be told about the patient's condition						0.042	0.998
Agree	53 (73.6%)	47 (73.4%)	66 (74.2%)	29 (72.5%)	195 (73.6%)	-	-
Disagree	19 (26.4%)	17 (26.6%)	23 (25.8%)	11 (27.5%)	70 (26.4%)	-	-
If laws allow abortion, doctors cannot refuse to perform an abortion						0.013	0.999
Agree	33 (45.8%)	29 (45.3%)	40 (44.9%)	18 (45.0%)	120 (45.3%)	-	-
Disagree	39 (54.2%)	35 (54.7%)	49 (55.1%)	22 (55.0%)	145 (54.7%)	-	-
If there is disagreement between patients/families regarding treatment, the healthcare professional's decision should be final						0.029	0.999
Agree	43 (59.7%)	39 (60.9%)	54 (60.7%)	24 (60.0%)	160 (60.4%)	-	-
Disagree	29 (40.3%)	25 (39.1%)	35 (39.3%)	16 (40.0%)	105 (39.6%)	-	-
If a patient wishes to die, he or she should be assisted in doing so no matter what their illness						0.022	0.999
Agree	11 (15.3%)	10 (15.6%)	13 (14.6%)	6 (15.0%)	40 (15.1%)	-	-
Disagree	61 (84.7%)	54 (84.4%)	76 (85.4%)	34 (85.0%)	225 (84.9%)	-	-
Given a situation, a male doctor needs to examine a female patient and a female attendant is not available; is it ethical to refuse the patient?						0.028	0.999
Agree	41 (56.9%)	36 (56.3%)	50 (56.2%)	23 (57.5%)	150 (56.6%)	-	-
Disagree	31 (43.1%)	28 (43.7%)	39 (43.8%)	17 (42.5%)	115 (43.4%)	-	-
Healthcare professionals must serve hard-to-reach areas and underserved populations?						0.043	0.998
Agree	63 (87.5%)	56 (87.5%)	77 (86.5%)	35 (87.5%)	231 (87.2%)	-	-
Disagree	9 (12.5%)	8 (12.5%)	12 (13.5%)	5 (12.5%)	34 (12.8%)	-	-

The overall mean awareness score for specific ethical issues was 27.4 ± 5.6 out of a maximum possible score of 40. No significant difference in mean awareness scores was observed between male and female participants; males had a mean score of 27.05 ± 5.5 compared with 27.89 ± 5.7 among females (p = 0.228). In contrast, participants with prior ethics training demonstrated significantly higher mean awareness scores than those without prior training, with mean scores of 28.50 ± 5.2 and 25.49 ± 5.7, respectively (p < 0.001).

Analysis of participants’ perspectives on various ethical issues showed that awareness increased with seniority, with consultants consistently getting the highest scores across all issues examined. For all ethical issues, the majority of participants classified the practice as unethical. The highest proportions were observed for discussing patient issues in public places, identified as unethical by 238 (89.8%) participants, followed by accepting fees for patient referrals by 205 (77.4%), prioritizing patients with influential recommendations by 203 (76.6%), and collecting donations from patients by 194 (73.2%). In contrast, accepting gifts from pharmaceutical companies was identified as unethical by the lowest proportion of participants, 139 (52.5%). Professional designation was significantly associated with responses to nine of the ten ethical issues (p < 0.05), whereas no significant association was observed for discussing patient issues in public places (p = 0.184) (Table 4).

**Table 4:** Healthcare professionals’ perspective regarding specific issues (n=265) N denotes the total number of participants in each group. Values are presented as n (%). Responses were scored as follows: Unethical = 4, Somewhat Unethical = 3, Uncertain = 2, and Ethical = 1. Associations were assessed using Pearson's χ² test. Cramer's V indicates the strength of association. Statistical significance was set at p < 0.05. *p < 0.05, **p < 0.01

Statement	House Officers (N=72)	Medical Officers (N=64)	PG Trainees (N=89)	Consultants (N=40)	Overall (N=265)	χ2 (p-value)	Cramer's V
Accepting gifts from patients						18.000 (0.035*)	0.151
Ethical	1 (1.4%)	3 (4.7%)	2 (2.2%)	0 (0.0%)	6 (2.3%)	-	
Uncertain	11 (15.3%)	16 (25.0%)	12 (13.5%)	1 (2.5%)	40 (15.1%)		
Somewhat Unethical	12 (16.7%)	12 (18.8%)	18 (20.2%)	4 (10.0%)	46 (17.4%)		
Unethical	48 (66.7%)	33 (51.6%)	57 (64.0%)	35 (87.5%)	173 (65.3%)		
Accepting gifts from pharmaceutical companies						26.783 (0.002**)	0.184
Ethical	13 (18.1%)	10 (15.6%)	2 (2.2%)	1 (2.5%)	26 (9.8%)	-	
Uncertain	11 (15.3%)	13 (20.3%)	14 (15.7%)	1 (2.5%)	39 (14.7%)		
Somewhat Unethical	14 (19.4%)	9 (14.1%)	24 (27.0%)	14 (35.0%)	61 (23.0%)		
Unethical	34 (47.2%)	32 (50.0%)	49 (55.1%)	24 (60.0%)	139 (52.5%)		
Accepting fee against referral to a specific doctor						17.321 (0.044*)	0.147
Ethical	2 (2.8%)	1 (1.6%)	4 (4.5%)	1 (2.5%)	8 (3.0%)	-	
Uncertain	20 (27.8%)	8 (12.5%)	8 (9.0%)	3 (7.5%)	39 (14.7%)		
Somewhat Unethical	4 (5.6%)	5 (7.8%)	4 (4.5%)	1 (2.5%)	14 (5.3%)		
Unethical	46 (63.9%)	50 (78.1%)	73 (82.0%)	36 (90.0%)	205 (77.4%)		
Advising the patient to buy a specific company's product						24.248 (0.004**)	0.175
Ethical	9 (12.5%)	12 (18.8%)	11 (12.4%)	0 (0.0%)	32 (12.1%)	-	
Uncertain	13 (18.1%)	6 (9.4%)	8 (9.0%)	1 (2.5%)	28 (10.6%)		
Somewhat Unethical	8 (11.1%)	6 (9.4%)	13 (14.6%)	13 (32.5%)	40 (15.1%)		
Unethical	42 (58.3%)	40 (62.5%)	57 (64.0%)	26 (65.0%)	165 (62.3%)		
Discussing patient issues in public places						12.550 (0.184)	0.126
Ethical	1 (1.4%)	3 (4.7%)	0 (0.0%)	0 (0.0%)	4 (1.5%)	-	
Uncertain	5 (6.9%)	0 (0.0%)	3 (3.4%)	1 (2.5%)	9 (3.4%)		
Somewhat Unethical	2 (2.8%)	4 (6.3%)	6 (6.7%)	2 (5.0%)	14 (5.3%)		
Unethical	64 (88.9%)	57 (89.1%)	80 (89.9%)	37 (92.5%)	238 (89.8%)		
Failing to disclose all significant medical errors to affected patients						23.366 (0.005**)	0.171
Ethical	10 (13.9%)	10 (15.6%)	7 (7.9%)	0 (0.0%)	27 (10.2%)	-	
Uncertain	15 (20.8%)	9 (14.1%)	9 (10.1%)	0 (0.0%)	33 (12.5%)		
Somewhat Unethical	8 (11.1%)	7 (10.9%)	13 (14.6%)	10 (25.0%)	38 (14.3%)		
Unethical	39 (54.2%)	38 (59.4%)	60 (67.4%)	30 (75.0%)	167 (63.0%)		
Not fully informing patients of the benefits and risks of a procedure or course of treatment						20.668 (0.014*)	0.161
Ethical	8 (11.1%)	9 (14.1%)	10 (11.2%)	0 (0.0%)	27 (10.2%)	-	
Uncertain	8 (11.1%)	8 (12.5%)	10 (11.2%)	0 (0.0%)	26 (9.8%)		
Somewhat Unethical	13 (18.1%)	5 (7.8%)	9 (10.1%)	12 (30.0%)	39 (14.7%)		
Unethical	43 (59.7%)	42 (65.6%)	60 (67.4%)	28 (70.0%)	173 (65.3%)		
Collecting donations from patients						19.896 (0.019*)	0.158
Ethical	13 (18.1%)	3 (4.7%)	4 (4.5%)	1 (2.5%)	21 (7.9%)	-	
Uncertain	6 (8.3%)	10 (15.6%)	10 (11.2%)	2 (5.0%)	28 (10.6%)		
Somewhat Unethical	4 (5.6%)	7 (10.9%)	9 (10.1%)	2 (5.0%)	22 (8.3%)		
Unethical	49 (68.1%)	43 (67.2%)	67 (75.3%)	35 (87.5%)	194 (73.2%)		
Patients with recommendations or influential references should be prioritized for treatment.						17.720 (0.039*)	0.149
Ethical	3 (4.2%)	4 (6.3%)	3 (3.4%)	0 (0.0%)	10 (3.8%)	-	
Uncertain	16 (22.2%)	4 (6.3%)	8 (9.0%)	2 (5.0%)	30 (11.3%)		
Somewhat Unethical	8 (11.1%)	5 (7.8%)	7 (7.9%)	2 (5.0%)	22 (8.3%)		
Unethical	45 (62.5%)	51 (79.7%)	71 (79.8%)	36 (90.0%)	203 (76.6%)		
The healthcare professional is performing his/her duty for personal gain						17.641 (0.040*)	0.149
Ethical	4 (5.6%)	5 (7.8%)	0 (0.0%)	0 (0.0%)	9 (3.4%)	-	
Uncertain	7 (9.7%)	5 (7.8%)	13 (14.6%)	0 (0.0%)	25 (9.4%)		
Somewhat Unethical	11 (15.3%)	8 (12.5%)	9 (10.1%)	7 (17.5%)	35 (13.2%)		
Unethical	50 (69.4%)	46 (71.9%)	67 (75.3%)	33 (82.5%)	196 (74.0%)		

## Discussion

In our study, the demographic distribution indicated that gender was not significantly associated with ethical awareness scores. In contrast, professional designation was significantly associated with ethical awareness levels, with higher awareness observed among more senior physicians. This trend is reflected in the proportions of participants achieving adequate overall awareness scores across the clinical hierarchy, which increased progressively from 54.2% among house officers and 60.9% among medical officers to 70.8% for postgraduate trainees, and peaking at 75.0% among consultants. These results aligned with the existing literature reporting an association between professional hierarchy and ethical awareness [14,15]. This incremental rise may reflect differences in professional experience, clinical exposure, and continued ethics learning acquired throughout medical practice.

Professional maturity may reflect not only accumulated clinical experience but also the cumulative effect of institutional training opportunities over time. This interpretation is further supported by our findings regarding prior ethics training. Participants who had received formal ethics education were more likely to demonstrate adequate ethical awareness than those without prior training (74.4% vs. 47.4%). This substantial difference suggests an association between ethics education and higher levels of ethical awareness, highlighting the potential value of structured ethics training in preparing healthcare professionals to recognize and address ethical challenges encountered in clinical practice. Consistent with these findings, a review by Farhan et al. recommended integrating the expertise of both clinicians and ethicists to strengthen medical ethics education and better bridge the gap between theoretical principles and bedside practice [16].

Regarding ethical dilemmas, the majority of participants (79.2%) agreed that children should not receive medical treatment without explicit parental consent. This finding aligns closely with previous research, which reported unanimous (100%) agreement among respondents [17]. The consistency observed across studies suggests that doctors generally regard parental involvement as a fundamental component of medical decision-making for minors. However, the slightly lower level of agreement observed in our study may reflect differing perspectives on situations in which parental consent may not be mandatory, such as medical emergencies or circumstances involving mature adolescents, where legal and ethical considerations are more complex [18].

Most participants (73.6%) agreed that close relatives should always be kept informed of a patient's medical condition. These results were in concurrence with previously conducted studies among undergraduate medical students [19,20]. This pattern may reflect the influence of regional cultural norms, where illness is often viewed as a family concern rather than solely an individual matter. In such settings, family involvement is commonly perceived as an important source of emotional, social, and practical support during illness.

The results regarding conscientious objection in abortion services revealed a notable lack of consensus among our participants. Only 45.3% of respondents agreed that a legally permitted abortion cannot be refused, indicating considerable variation in ethical perspectives within the cohort. This contrasts with previous research, where a clear majority of participants asserted a stronger right to refuse the procedure [10]. This divergence suggests that the professional perspective on balancing legal obligations with personal conscience remains highly context-dependent. A significant factor in this discrepancy may be the geographical and cultural background of the study populations. The observed difference may reflect regional variations in cultural, religious, and ethical perspectives regarding abortion. The more divided responses in our study may also indicate differing views on balancing personal conscience with professional obligations to provide legally permitted healthcare services. These findings suggest that ethical perspectives on conscientious objection may vary according to the sociocultural context in which healthcare professionals practice, underscoring the importance of considering local context when developing and implementing medical ethics education and policy.

In response to the statement that patients should be assisted in dying regardless of their illness, the majority of participants expressed strong disagreement. These findings are consistent with previous studies and may reflect the influence of professional ethics, legal prohibitions, and prevailing religious and cultural values [21].

When analyzing specific ethical issues, our findings reveal that ethical awareness varied considerably according to the nature of the scenario presented. Participants demonstrated a strong, unified consensus on overt ethical violations; however, this consensus was less pronounced for scenarios involving conflicts of interest, systemic biases, or more complex ethical decisions.

The most concerning findings related to the acceptance of gifts from pharmaceutical companies and patients or accepting fees for specialist referrals. Notably, only 52.5% of participants identified accepting gifts from pharmaceutical companies as unethical, highlighting a potential area of vulnerability in ethical awareness. These findings are consistent with those reported in Lebanon, where 70.4% of physicians reported receiving gifts from pharmaceutical representatives, and only 40% considered the practice unethical [22].

However, a more detailed analysis of our data revealed a clear hierarchical pattern. Although the overall sample showed relatively lower recognition of the ethical concerns surrounding these financial relationships, this proportion increased progressively with professional designation and was highest among consultants. A similar trend was observed across related conflicts of interest, including the acceptance of gifts from patients and fees for specialist referrals. This pattern suggests that junior clinicians may underestimate the ethical implications that industry marketing and financial incentives have on clinical objectivity [23]. Given the widespread interaction between healthcare professionals and the pharmaceutical industry, ethics education should explicitly address potential conflicts of interest and emphasize the importance of maintaining professional independence and patient trust [24].

A similar linear relationship with professional designation was observed regarding the prioritization of patients who present with influential references or recommendations. The proportion of participants considering this practice unethical increased progressively with clinical rank, with house officers demonstrating comparatively lower recognition of its ethical implications. In resource-constrained healthcare environments, junior clinicians often work on the front lines with limited institutional authority and may therefore find it more difficult to resist social or administrative pressures [25]. This may contribute to greater acceptance of requests based on personal influence, a practice that conflicts with the ethical principle of justice, which requires the fair and equitable allocation of healthcare resources [2].

In contrast to these hierarchy-related differences, attitudes toward patient confidentiality showed remarkable consistency across professional designations. Overall, 89.8% of participants identified discussing patient cases in public spaces as unethical, and this was the only ethical scenario that showed no statistically significant association with professional designation. This strong consensus suggests that the importance of maintaining patient confidentiality is widely recognized across all levels of medical practice [18,26]. Despite the demanding and fast-paced environment of tertiary care hospitals, respect for patient privacy appears to be a well-established ethical norm among the physicians included in our study.

Additionally, the study explored attitudes towards transparency in clinical communication, including both the disclosure of medical errors and the communication of treatment risks and benefits. Overall, 167 (63.0%) participants considered failing to disclose medical errors unethical, while an additional 38 (14.3%) regarded it as somewhat unethical. A similar pattern was observed for informed consent, with 173 (65.3%) participants recognizing that failing to fully disclose the risks and benefits of a procedure constitutes an ethical concern. Our findings are consistent with previous literature emphasizing the ethical importance of transparency in clinical practice. In another study, approximately 87% of physicians supported disclosing medical errors resulting in major harm, while 76% also favored disclosure when errors caused only minor harm [27].

Although the majority of participants in our study demonstrated adequate ethical awareness, the overall level of consensus was lower than expected for a professional healthcare setting. Similar concerns have been reported in the literature, with studies identifying important gaps in physicians' knowledge of foundational ethical principles. For example, one study reported that 96% of physicians were unaware of the details of the Nuremberg Code or the Declaration of Helsinki [28].

Taken together, these findings suggest that although many physicians recognize fundamental ethical principles, familiarity with the formal ethical frameworks underpinning medical practice may remain limited. These observations underscore the importance of strengthening ethics education throughout medical training to promote a more comprehensive understanding and consistent application of ethical principles in clinical practice.

Our study recommends that structured and mandatory ethics training programs be implemented for all doctors, with particular emphasis on house officers and medical officers who demonstrated comparatively lower awareness levels. Ethics education should be more comprehensively integrated into the undergraduate medical curriculum, focusing on practical application through case-based learning and real-life scenarios to build a strong ethical foundation early in training. Additionally, healthcare institutions should organize regular continuing professional development programs, including workshops and seminars, to ensure ongoing reinforcement of ethical knowledge. The development and enforcement of clear institutional ethical guidelines and protocols are also essential to support informed decision-making in clinical practice. Consideration should also be given to the influence of cultural and societal norms and beliefs on ethical decision-making, with training programs incorporating culturally sensitive approaches to ensure ethical principles are applied effectively within the local context.

Some limitations must be acknowledged when interpreting the findings of this study. A cross-sectional study design precludes the ability to track how these attitudes might evolve over a career or in response to changing institutional policies. A sample restricted to doctors within a single hospital may constrain the generalizability of the findings across different healthcare professions or diverse institutional settings. Furthermore, the study’s reliance on self-reported questionnaires introduces the potential for social desirability bias, as participants may have provided responses deemed professionally correct rather than reflecting their genuine perceptions. This was mitigated by conducting the use of anonymous data collection, which fostered an environment for honest disclosure. It is also important to note that this study measures ethical awareness and perceptions rather than actual clinical behaviors, meaning the findings may not fully capture how these practitioners would act when faced with these dilemmas in real-world practice.

## Conclusions

While a majority of participants demonstrated adequate awareness, notable gaps were observed, particularly among junior doctors and those without formal ethics training. These findings indicate the role of professional experience and structured education in shaping ethical understanding and emphasize the need for mandatory, structured, and practice-oriented ethics training within undergraduate and postgraduate medical education. This training should also be integrated into clinical rotations through case-based workshops and evaluated through practical assessments such as Objective Structured Clinical Examinations (OSCEs), with particular emphasis on junior doctors and other healthcare professionals lacking prior ethics training.

## Appendices

Appendix 1

**Table 5:** Questionnaire Questionnaire adapted from Ateeb et al. [10] and based on the validated instrument developed by Al-Busaidi et al. [11]. Minor modifications were made to suit the study context. The original instrument was published under the Creative Commons Attribution (CC BY 4.0) license.

Your participation in this study is completely voluntary, and all your responses will be kept confidential. We hope you will support this study by providing valuable information.																																
Do you agree to participate?	Yes															No																
Section A: General Information																																
Gender	Male															Female																
Age	20-30									31-40														41 or above								
Do you regularly work with patients at the hospital?	Yes															No																
Department of posting	Medicine & Allied						Surgery & Allied									Gynecology and Obstetrics											Other					
Ethnicity / Cultural Background	Punjabi		Pathan								Saraiki							Sindhi					Baloch								Other	
Designation of your job	Consultant				Medical Officer								House Officer						Post-Graduate Trainee											Other		
Country of professional education	Pakistan														Foreign																	
Professional job experience	< 5 years								5-10 years													> 10 years										
During professional study, how many credit hours were there for ethics in your curriculum?	1-5 hours					6-10 hours									> 10 hours											Did not study						
Do you know the four principles of medical ethics?	Yes														No																	
How do you rate the relevance of the teaching/training of medical ethics to your practice now? (1 = weak relevance, 10 = strong relevance)	1	2		3				4						5			6				7				8			9				10
Has your organization developed a written code of ethics that outlines what is considered ethical?	Yes														No																	
Section B: Ethics in Healthcare Practice																																
How often do you encounter an ethical situation in your practice?	Rare (once a year)					Occasional (once every 6 months)									Often (once every month)											Frequent (once every week)						
How often do you find an answer to your question on ethical issues?	Rare (once a year)					Occasional (once every 6 months)									Often (once every month)											Frequent (once every week)						
How often do you observe an unethical decision in your practice?	Rare (once a year)					Occasional (once every 6 months)									Often (once every month)											Frequent (once every week)						
In what areas do you encounter ethical issues? (Check all that apply)	Religion		Law								Financial							Conflict of interest					Tradition and values								Other	
Where do you usually look for an answer to your ethical question? (Check all that apply)	Books				Internet								Friends							Senior colleagues									Elsewhere			
What mostly stops you from unethical practice? (Check all that apply)	Religion				Law								Ethics teachings							Tradition and values									Other			
Section C: Ethical Dilemmas																																
Children should not be treated without the consent of their parents.																				Agree							Disagree					
Close relatives should be told about patient’s condition.																				Agree							Disagree					
If laws allow abortion, doctors cannot refuse to do an abortion.																				Agree							Disagree					
If there is a disagreement between patients/families about treatment, the healthcare professional’s decision should be final.																				Agree							Disagree					
If a patient wishes to die, he or she should be assisted in doing so no matter what their illness.																				Agree							Disagree					
Given a situation where a male doctor needs to examine a female patient and a female attendant is not available, is it ethical to refuse the patient?																				Agree							Disagree					
Healthcare professionals must serve hard-to-reach and under-served populations.																				Agree							Disagree					
Section D: Specific Issues in Medical Ethics																																
Accepting gifts from patients	Ethical		Uncertain									Somewhat unethical														Unethical						
Accepting gifts from pharmaceutical companies.	Ethical		Uncertain									Somewhat unethical														Unethical						
Accepting fee against referral to a specific doctor.	Ethical		Uncertain									Somewhat unethical														Unethical						
Advising the patient to buy a specific company’s product.	Ethical		Uncertain									Somewhat unethical														Unethical						
Discussing patient issues in public places.	Ethical		Uncertain									Somewhat unethical														Unethical						
Failing to disclose all significant medical errors to affected patient.	Ethical		Uncertain									Somewhat unethical														Unethical						
Not fully informing patients of the benefits and risks of a procedure or course of treatment.	Ethical		Uncertain									Somewhat unethical														Unethical						
Collecting donations from patients.	Ethical		Uncertain									Somewhat unethical														Unethical						
Prioritizing patients with recommendations or influential references.	Ethical		Uncertain									Somewhat unethical														Unethical						
Performing duties mainly for personal monetary benefit.	Ethical		Uncertain									Somewhat unethical														Unethical						
